# Combined Experimental and Theoretical Approach to the Electronic and Magnetic Properties of Cu-Doped LaMnO_3_ Perovskites

**DOI:** 10.1021/acs.jpcc.4c06256

**Published:** 2024-12-23

**Authors:** Josef M. Gallmetzer, Felix R. S. Purtscher, Jakob Gamper, Asghar Mohammadi, Ralf Feyerherm, Wiebke Riedel, Simon Penner, Thomas S. Hofer

**Affiliations:** † Institute of General, Inorganic and Theoretical Chemistry, University of Innsbruck,Innrain 80-82, 6020 Innsbruck, Austria; ‡ Institute of Physical Chemistry, University of Innsbruck, Innrain 52c, 6020 Innsbruck, Austria; § Institute Quantum Phenomena in Novel Materials, Helmholtz-Zentrum Berlin für Materialien und Energie GmbH, Hahn-Meitner-Platz 1, 14109 Berlin, Germany; ∥ Institute of Physical and Theoretical Chemistry, Free University of Berlin, Arnimallee 22, 14195 Berlin, Germany

## Abstract

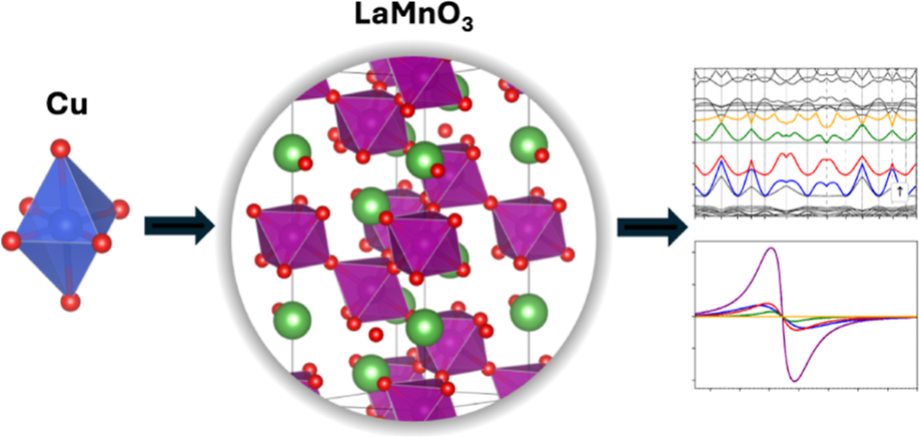

Cu-doped LaCu_*x*_Mn_1–*x*_O_3_ perovskites have been used as a model system for a joint experimental and theoretical assessment of the influence of the Cu doping level on the structural, electronic, and magnetic properties. The different Cu-doped phases LaCu_0.3_Mn_0.7_O_3_ (LCM37), LaCu_0.5_Mn_0.5_O_3_ (LCM55), and LaCu_0.7_Mn_0.3_O_3_ (LCM73) including the respective Cu- and Mn-free benchmark materials La_2_CuO_4_ (LC) and LaMnO_3_ (LM) have been studied by magnetization measurements and electronic paramagnetic resonance. Ferromagnetic behavior was detected for pure LM and all Cu-doped perovskites, whereas antiferromagnetic behavior was revealed for La_2_CuO_4_. Generally, an increased antiferromagnetic contribution was shown for higher Cu doping levels. Equally, magnetization was highlighted to decrease with increasing Cu content. Sophisticated hybrid density functional theory calculations of the electronic and magnetic properties using defect-free, idealized Cu-doped model structures agree well with the experimental results. The findings reveal that copper incorporation influences both the electronic conductivity and the magnetic properties. Notably, the materials exhibit a tunable degree of half-metallicity and significant electronic spin polarization, establishing them as promising candidates for advanced technological applications in spintronics and catalysis. The insights gained from this study contribute to a broader understanding of perovskite materials and their versatile applications.

## 1 Introduction

Perovskites with the general formula ABX_3_ are a promising class of materials with a wide range of applications,^1−3^ from high-efficiency solar cells^4,5^ and electrochemistry^6^ to heterogeneous catalysis^7^ and magnetism.^8^ A-site ions are typically divalent alkaline earth [e.g., Sr(II) or Ca(II)] or trivalent lanthanide [La(III) and Sc(III)] ions. The B sites are usually occupied by multivalent transition metal ions capable of establishing a redox couple to ensure charge neutrality. X-site ions are typically oxygen atoms or halides.

A key parameter of the use of perovskites is the ability to tune their physicochemical or catalytic properties by doping at the A or B site and to accordingly impact the oxygen vacancy concentration.^9^ Especially the latter is strongly dependent on the synthesis conditions, oxygen partial pressures, or humidity levels. Equally, the perovskite structure can typically exist under a multitude of polymorphic modifications as a function of those experimental conditions. An archetypical model system in which these features are well-illustrated is LaMnO_3_. Phase- and structure-pure LaMnO_3_ already exists in three polymorphic forms: orthorhombic at room temperature, cubic between room temperature and 1010 K, and rhombohedral above 1010 K.^10−14^

LaMnO_3_ has been widely screened for applications in transport processes and magnetism such as colossal magnetoresistance. In due course, photocatalytic applications, use as pseudocapacitors and heterogeneous or electrocatalysts, are reported.^15−25^ From a theoretical viewpoint, Koriba et al.^15^ have provided input into the structural, magnetic, electronic, and mechanical properties of different polymorphic LaMnO_3_ compounds. Additionally, Ma et al.^26^ specifically investigated the potential of LaMnO_3_ in spintronic applications. They showed that LaMnO_3_ exhibits 100% electronic spin polarization and linear band crossings (i.e., Dirac cones)^27^ at the Fermi level, both of which are critical for developing spintronic devices with zero energy dissipation. These findings underscore the material’s promise for energy-efficient spintronic technologies.

As the dominant steering parameter of perovskite properties is the variation of the doping level at the respective A and B site, control of the redox couple at the B site is of utmost importance.^9^ With respect to doped LaMnO_3_ perovskites, this is usually ensured by the presence of an Mn(III)/Mn(IV) redox pair. In pure LaMnO_3_, the manganese ions are only present in oxidation state +3, whereas doping with other ions or the presence of defects may lead to the formation of Mn(IV) in the perovskite lattice.^24,25,28,29^ The consequence of doping, therefore, is the modification of the Mn(III)–O–Mn(IV) network^30−33^ and the direct steering of the associated structural, electronic, catalytic, or magnetic properties.^11,30,34−41^

A particularly promising dopant material, where these features can be directly assessed, is Cu.^30,34−41^ Cu as one the most widely used dopant material causes the formation of an additional redox couple Cu(II)/Cu(III).^42^ This induces a change in the conduction mechanism by an additional interaction between the Mn and the doping Cu ion, affecting the interaction between Mn(III) and Mn(IV) in a similar way.^31−33,43^ Doping LaMnO_3_ with Cu can also modify its electronic spin polarization and properties, enhancing its suitability for spintronic applications. This tunability through Cu doping allows for precise control of the electronic and magnetic characteristics, rendering LaCu_*x*_Mn_1–*x*_O_3_ as a versatile and highly promising candidate for advanced spintronic devices. We have also shown previously how the Cu/Mn ratio within the LaCu_*x*_Mn_1–*x*_O_3_ lattice critically influences the catalytic performance in the selective catalytic reduction of NO by CO.^44^ We demonstrated that an increase in the Cu content enhances the number of oxygen vacancies, which are critical for the catalytic reduction and regeneration process. Additionally, we revealed that optimal steering by Cu maximizes surface reactivity while maintaining the structural integrity under high-temperature and operation conditions. Similar effects of Cu-doped LaMnO_3_ catalysts have been obtained in NOx reduction,^45^ styrene combustion,^46^ methanol synthesis,^47^ catalytic combustion of carbon monoxide^48^ or methane,^49^ chemical looping methane steam reforming,^50^ or carbon monoxide oxidation.^51^

Doped LaCu_*x*_Mn_1–*x*_O_3_ materials also offer the possibility of studying the effect of doping on the structural integrity. Several studies indicate that structure- and phase-pure LaCu_*x*_Mn_1–*x*_O_3_ can only be obtained for compositions between 0 ≤ *x* ≤ 0.6.^30,34−41^ Higher Cu levels unequivocally cause the formation of either La_2_CuO_4_ or CuO, which are a direct result of exceeding the Cu doping limit of the parent LaMnO_3_ structure. We have also recently highlighted how the delicate balance between composition, final calcination temperature, and activation in the NO + CO reaction mixture can be beneficially tuned to engineer the Cu-LaCu_*x*_Mn_1–*x*_O_3_ interface to optimum reactivity.^44^ Similarly, Cu doping of LaMnO_3_ has a pronounced effect on other perovskite properties, such as ferromagnetic (FM) insulating behavior, domain wall pinning,^52^ or large magnetocaloric effects.^53^

Despite the large body of data compiled for pure LaMnO_3_ and Cu-doped LaMnO_3_ materials, a correlative experimental and theoretical study on the effect of Cu doping on the properties of LaMnO_3_ perovskite materials is still missing. To close this knowledge gap, this work provides input into the correlation of experimentally determined magnetic properties and theoretical predictions from model Cu-doped LaMnO_3_ structures. We provide room temperature continuous wave electronic paramagnetic resonance (EPR) and temperature- and magnetic-field-dependent magnetization measurements and compare them to theoretical results obtained for idealized, defect-free systems to elucidate the effect of Cu addition on their electronic and magnetic properties. A prerequisite to provide such theoretical studies is the model access to perovskite structures, which closely resemble those synthesized for the experimental investigations. Hence, the first objective of the work is to derive a successful theoretical doping strategy based on experimentally obtained composition-dependent X-ray diffraction patterns. For our studies, we have selected five different stoichiometries: the benchmark Cu- and Mn-free LaMnO_3_ and La_2_CuO_4_ structures and three Cu-doped compositions resembling the experimental LaCu_0.3_Mn_0.7_O_3_ (LCM37), LaCu_0.5_Mn_0.5_O_3_ (LCM55), and LaCu_0.7_Mn_0.3_O_3_ (LCM73) stoichiometries. Second, based on the modeled structures, we highlight that successful theoretical predictions on the magnetic and electronic properties can be derived: the observed FM behavior at low temperatures, the reduction in magnetic moment as a function of Cu doping, and the magnetic resonance measurements are in excellent agreement with theoretical calculations.

## 2 Methods

### 2.1 Experimental Details

The synthesis of the LaCu_*x*_Mn_1–*x*_O_3_ samples has been conducted by a sol–gel approach, as described in a previous study.^44^

Temperature- and magnetic-field-dependent magnetization measurements have been carried out on a Quantum Design Physical Properties Measurement System. Small amounts of sample (10–15 mg) were filled into plastic capsules and mounted onto brass sample holders. For each sample, the temperature-dependent magnetization between 2 and 300 K has been measured after zero-field cooling (zfc) from 300 to 2 K and after field cooling (fc) at an external field of 100 mT. At 2 and 300 K, full hysteresis curves have been measured up to 5 and 1 T, respectively. Room temperature continuous wave EPR (cw-EPR) measurements at X-band (9.86 GHz) frequencies have been conducted with a Bruker B-ER420 spectrometer upgraded with a Bruker ECS 041XG microwave bridge and a lock-in amplifier (Bruker ER023M) using a Bruker TE_102_ resonator applying a modulation amplitude of 5 G, a modulation frequency of 100 kHz, and an attenuation of 20 dB for the microwave bridge. The samples have been measured in quartz tubes of 2.9 mm outer diameter with a filling height of approximately 9 mm containing approximately 7–20 mg of the powdered sample. All spectra are background-corrected, taking an empty quartz tube as reference and normalized to the sample mass and quality factor of the resonator for the specific measurement.

Powder X-ray diffraction (PXRD) patterns of the samples have been recorded using a Rigaku SmartLab-SE instrument in parallel beam setting and reflection mode (Co K_α_, λ = 1.789 Å) using a D/teX Ultra 250 compound silicon strip 1D detector. The patterns have been recorded in a range of 2Θ = 5° to 80° with a step width of 0.005°. Further details on the characterization of the samples can be found in Supporting Information Section S1.

### 2.2 Computational Details

The calculations have been performed using the solid-state density functional theory (DFT) code Crystal23,^54^ employing the hybrid functional HSEsol^55^ together with the pob-DZVP-rev2 basis set^56^ for Cu, Mn, and O, and the pob-TZVP-rev2 basis set^57^ incorporating an effective core potential for La. HSEsol is a range-separated hybrid functional based on the popular PBE GGA functional,^58^ that was especially developed to enable an improved description of solid-state systems.

To ensure an accurate treatment, structural and unit cell optimizations were conducted with a shrinking factor of 16. The convergence criteria for energy and forces have been set to 10^–7^ hartree and 4.5 × 10^–4^ hartree bohr^–1^, respectively.

To compare the resulting theoretical and experimental structures, PXRD patterns have been generated using RIETAN-FP^59^ (Co K_α_, λ = 1.7902 Å) as implemented in the VESTA program.^60^ Additionally, the electronic band structure and the associated total and projected densities of states (TDOS/PDOS) of the optimized geometries have been calculated using Crystal23. The high symmetry paths have been determined using an improved symmetry-based approach^61^ based on the Setyawan–Curtarolo^62^ path, as implemented in the pymatgen software package.^63^

Furthermore, the electronic distribution of the compounds has been calculated using the Mulliken population analysis^64^ implemented in Crystal23. From the respective occupations, individual charges and spin states can be derived, which are directly linked to the magnetic properties of the materials.

## 3 Results and Discussion

### 3.1 Structural and Magnetic Properties of Experimental Samples

In a previous work, we have structurally and compositionally characterized the samples that were used for the magnetic experiments and as base structures for theoretical modeling.^44^

**Figure 1:**
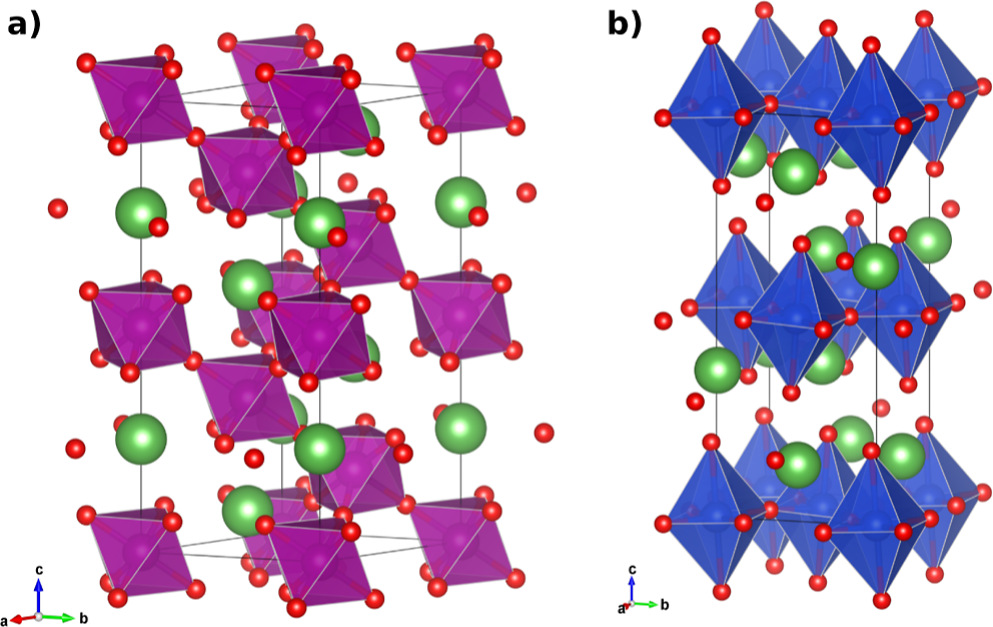
Crystal structure of LaMnO_3_ (a) and La_2_CuO_4_ (b) in the rhombohedral *R*3̅*c*^65^ and orthorhombic *Bmab*(66) space groups, respectively. The La and O atoms are depicted in green and red. The MnO_6_ and CuO_6_ octahedra are highlighted in purple and blue, respectively.

Next to mixed compounds containing both Mn and Cu, pure LaMnO_3_ (LM) and pure La_2_CuO_4_ (LC) were synthesized. PXRD measurements have shown that the LaMnO_3_ perovskite crystallizes in space group *R*3̅*c* (167),^65^ while La_2_CuO_4_ forms a spinel structure in space group *Bmab* (64)^66^ (see Figures 1 and S1). For the mixed compounds with Cu doping levels of *x* = 0.37, 0.55, and 0.73 (denoted as LCM37, LCM55, and LCM73), diffraction patterns (Figure S2) indicated that the doped compounds crystallize in the orthorhombic space group *Pnma* (62). Higher Cu doping levels result in the formation of La_2_CuO_4_ and CuO parasitic structures, as detected for LCM73, in agreement with the literature.^67^ These structures arise if the Cu doping limit of the parent LaMnO_3_ structure is exceeded.^68−70^

Elemental analysis by inductively coupled plasma spectroscopy (Table S1) confirms that the stoichiometry closely matches the nominal composition from the synthesis. Specific surface area measurements by Brunauer–Emmett–Teller (BET) indicate that Cu doping has no substantial influence on the specific surface area. In addition, we have previously also provided surface compositional analysis by X-ray photoelectron spectroscopy (XPS). As expected for perovskites with La at the A site,^71,72^ the surface of all Cu-doped samples is enriched in La, i.e., the surface composition strongly deviates from the bulk composition. As for the surface-bound oxidation states of Cu and Mn, previous XPS analysis of the Cu 2p spectra essentially indicated predominantly Cu(II), with a minor contribution of Cu (I/0), at least for LCM37 and LCM55.^73^ The Mn 2p spectra indicate the presence of Mn(III) and Mn(IV).^44^ The determination of the respective bulk oxidation states of Cu and Mn proved challenging due to the presence of overlapping parasitic structures with Cu(II) contribution (CuO and La_2_CuO_4_), especially at higher Cu contents. The magnetic measurements discussed below at least indicate a mix of Mn(III)/Mn(IV), as well as Cu(II)/Cu(I).

**Figure 2:**
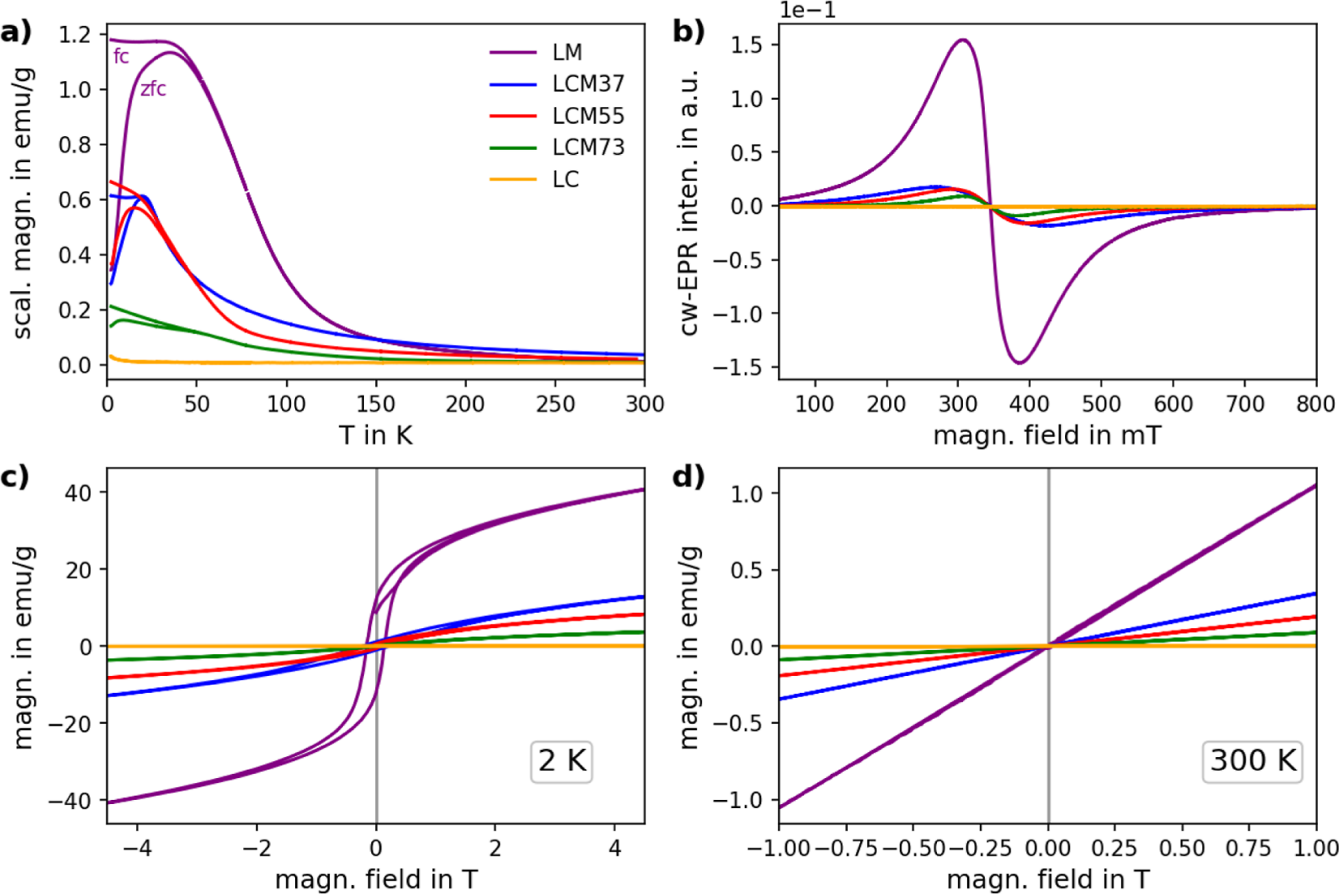
(a) Temperature dependence of magnetization (fc and zfc) of the different samples measured at a magnetic field of 100 mT. LM is scaled by a factor of 0.1. (b) Room temperature cw-EPR measurements of LM, LC, and LCM samples with varying Cu content performed at 9.64 GHz. All spectra are normalized to the sample mass. Magnetization curves measured at (c) 2 K and (d) room temperature of different LCM samples as well as LM and LC references.

The magnetic properties of the samples were investigated by temperature- and field-dependent magnetization measurements as well as by cw-EPR spectroscopy conducted at room temperature (Figure 2). For the LC sample, the experimental observation of negligible signals in both magnetization and EPR measurements is consistent with antiferromagnetic (AFM) ordering with a Nèel temperature >300 K.^74^ For the LM sample, FM behavior is detected with transition temperatures of approximately 75 K and a significantly less pronounced transition around 215 K suggesting the presence of a minor cubic phase.^75^ The transition temperatures are slightly higher than those reported by Jonker but differ notably from those by Porta et al. and Malavasi et al., who reported values of 160 and 147 K highlighting how strongly the magnetic properties are affected by preparation conditions.^67,75,76^ The Cu-containing LCM samples also exhibit a FM behavior. The transition temperatures for LCM37, LCM55, and LCM73 are approximately 70, 50, and 45 K, respectively. The temperatures and their decrease with increasing Cu content are overall in fair agreement with a previous study by Porta et al. on similar samples.^67^ For La_2_CuMnO_6_, it was previously shown that the system exhibits a combination of AFM and FM interactions, showing at low temperatures a maximum in the zfc curves and high coercivities in field-dependent measurements.^34,77^ The samples investigated here show similarly a maximum in the zfc curves and high coercivities at low temperatures exhibiting clear changes with increasing Cu content; i.e., the maximum temperature and coercivity are decreasing with increasing Cu content. The dip in the zfc curves associated with AFM is most pronounced for the sample with the highest Cu content. Note that no clear indication for an AFM CuO phase was detected for the samples with high Cu content, presumably due to overall low amounts of this minority phase. With increasing Cu content, also the magnetization at low temperatures strongly decreases, as measured in field-dependent measurements. Similarly, the magnetization at high temperatures and, accordingly, the effective magnetic moment in the paramagnetic regime decrease for higher Cu contents. The effective magnetic moment of the Cu-containing samples is comparable with values reported by Porta et al. but differs more strongly for the LM sample.^67^ Moreover, it deviates from the theoretically expected spin-only values, assuming contributions from Mn(III), Mn(IV), and Cu(I). cw-EPR measurements conducted at room temperature show for the LM and LCM samples a broad signal around *g* = 2 which decreases in intensity with increasing Cu content. The intensity decrease for higher Cu contents roughly persists when considering the Curie temperatures of the different samples. For LM samples, such broad EPR signals have previously been attributed to Mn(IV) species or interacting Mn(III)–Mn(IV) species.^78−80^ An assignment to just Mn(IV) [or just Cu(II)] species is clearly not meaningful, as the signal should increase for higher Cu content, which is not observed. EPR rather probes, even at room temperature, a strongly interacting spin system that is significantly affected by the addition of Cu.

Thus, the results clearly demonstrate that the incorporation of Cu crucially affects the magnetic properties. Moreover, they also emphasize the importance of preparation conditions and the associated number of defects for magnetic properties. While the prepared samples are clearly not defect-free, ideal compounds as assumed in theoretical calculations, this set of samples prepared under otherwise identical conditions may serve for a more consistent comparison to theory results for an improved atomistic understanding of the effect of Cu addition.

Based on the outlined experimental results, we attempted to provide a theoretical confirmation for the observed magnetic properties of the perovskite phases with different Cu doping levels. For the most detailed and clear link between experiments and theory, a sophisticated approach to theoretical model structures that reproduce the observed experimental structure characterization is imperative. This approach is in detail described in the following Section 3.2. The match between experiment and theory is a prerequisite for a reliable theoretical prediction of the magnetic properties for the Cu-doped LaMnO_3_ perovskites, see Section 3.4, and a correct interpretation of the experimental results in the preceding sections.

### 3.2 Theoretical Doping Strategy

**Figure 3:**
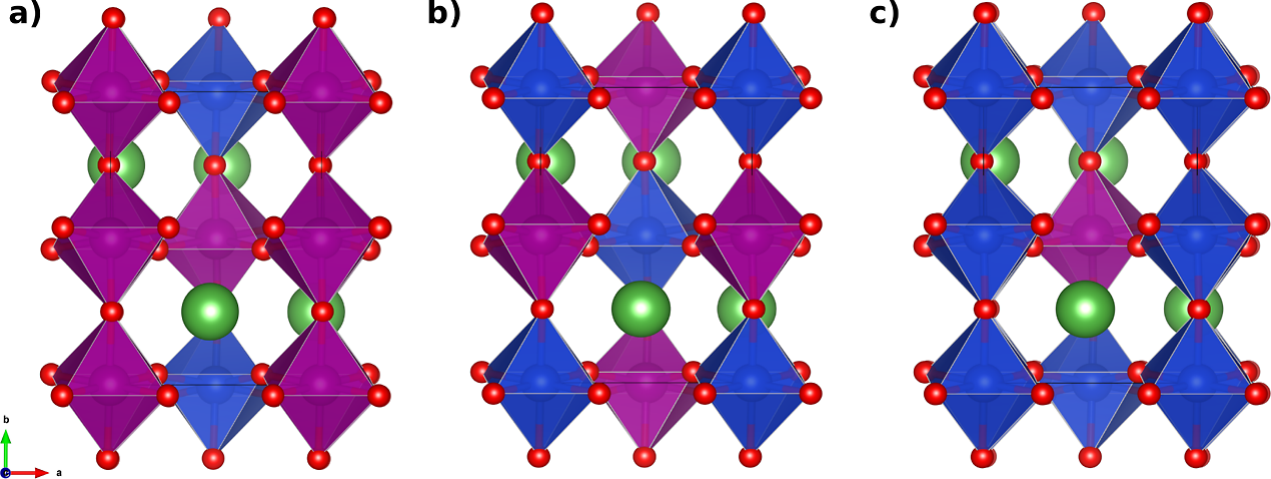
Crystal structure of Cu-doped LaMnO_3_ exhibits an orthorhombic *Pnma* symmetry.^40^ Specifically, the structures are (a) La_4_Cu_1_Mn_3_O_12_ (LCM37), (b) La_4_Cu_2_Mn_2_O_12_ (LCM55), and (c) La_4_Cu_3_Mn_1_O_12_ (LCM73). In the case of LCM55, Cu atoms occupy the Mn sites at positions 2 and 4 within the unit cell, as detailed in Table S2, resulting in alternating Cu–O–Mn chains along the *b* axis. The La and O atoms are depicted in green and red, and the MnO_6_ and CuO_6_ octahedra are highlighted in purple and blue, respectively.

Diffraction patterns of the experimental data have indicated that the doped compounds crystallize in orthorhombic space group *Pnma* (62). Accordingly, the doped LaCu_*x*_Mn_1–*x*_O_3_ systems have been constructed based on the orthorhombic structure of LaMnO_3_ doped with Cu, as reported by Petrov.^40^ The new compounds have been studied with Cu doping levels of *x* = 1/4, 1/2, and 3/4 in the Mn crystal site, resulting in La_4_Cu_1_Mn_3_O_12_ (LCM37), La_4_Cu_2_Mn_2_O_12_ (LCM55), and La_4_Cu_3_Mn_1_O_12_ (LCM73) systems, respectively. This has been achieved by systematically substituting Mn with Cu according to the doping level, while maintaining fixed La and O site occupations. Due to the varying doping levels, the unit cell of the LCM compounds has been expanded to contain four formula units. Considering the different possible configurations of these systems, it has been determined that for both LCM37 and LCM73, the possible configuration isomers are invariant, and thus, only one isomer has been studied for each doping level. However, for the LCM55 system, three distinct configuration isomers have been identified, as the Cu atoms can occupy different positions within the unit cell, resulting in different Cu–O–Mn chains. The crystal structures of the LCM compounds are depicted in Figure 3.

It is important to note that the experimentally determined dopant concentrations deviate from the theoretical values, with Cu doping levels of *x* = 0.37, 0.55, and 0.73 for LCM37, LCM55, and LCM73, respectively.

### 3.3 Structural Properties

Following the completion of the geometry optimization process, the structural properties of the compounds have been analyzed. The PXRD patterns of the systems have been compared to the experimental data, as illustrated in Figures S1 and S2. The theoretical reflections for both pristine LM and LC align well with the experimental data, validating the reliability of the calculations. However, the LC sample exhibits some phase impurities, which have been identified as a CuO phase^81^ also present in the experimental data of LCM73 (Figure S2). Higher Cu doping levels result in the formation of La_2_CuO_4_ and CuO parasitic structures. These structures arise if the Cu doping limit of the parent LaMnO_3_ structure is exceeded.^68−70^

As previously mentioned, different configurations of isomers have been identified for the LCM55 system. Table S2 presents these configuration isomers and their respective energy differences. It is evident that the different isomers exhibit remarkably similar minimum energies. The lowest-energy isomer is characterized by Cu atoms occupying the Mn sites at positions 2 and 4 (Table S2) within the unit cell, resulting in the formation of an alternating Cu–O–Mn chain along the *b* axis, as shown in Figure 3. This isomer has been used for further calculation of the electronic and magnetic properties of the compounds.

The resulting cell parameters, following the geometry optimization process, are detailed in Section S4. Table S3 shows the lattice parameters of the LCM structures. The optimized geometries exhibit slight deviations, which can be attributed to the varying doping levels of Cu. The largest overall deviation has been observed for LCM55, with a 1.5% deviation in the *a* lattice parameter compared with the experimental data. Additionally, a slight offset of the lattice angles has been noted, with the largest deviation identified as 0.4° from the experimental 90°.

Although the overall optimization of LM is in good agreement with the literature, the optimization of LC shows larger deviations (Tables S4 and S5). The deviation in the *c* lattice parameter of LC is approximately 2% compared to the experimental data, while the deviation in the *a* lattice parameter is approximately 1%.

Table 1 presents the bond lengths of Mn–O, Cu–O, and La–O for the different systems. The Mn–O bond lengths are in the range of 1.941–1.989 Å, indicating that no major Jahn–Teller (JT) distortion is present in LM and LCM. Conversely, LC shows a clear JT *z* elongation, evidenced by the distances of 1.883 and 2.368 Å between the equatorial Cu–O2 and axial Cu–O1, respectively. Despite LC exhibiting strong *z* elongation, no significant JT effect was observed in the Cu-doped LCM systems. This effect may be caused by the structure in the *Pnma* space group of the doped compounds being less prone to JT distortions compared to the structure of LC (*Bmab* space group).^26^ However, pristine LaMnO_3_ has been reported to exhibit a JT distortion in the *Pnma* space group,^82,83^ which is not observed in the doped systems. Due to the higher local symmetry of the non-JT-distorted octahedrons, the FM ground state is more stable than its AFM counterpart, in agreement with experimental data. A study by Ma et al.^26^ has shown that the LM compound exhibits a JT distortion in the structure derived from the *Pnma* space group, which is not observed in the doped compounds. Additionally, the findings of Ma et al.^26^ suggest that the JT distortion of LM in the rhombohedral structure (*R*3̅*c* space group) is suppressed, which leads to the FM state.

**Table 1:** Average Metal Oxide Bond Lengths of the LM, LC, and LMC Perovskites for Different Cu Concentrations *x*^a^

atom	*x*	Mn–O1/Å	Mn–O2/Å	Cu–O1/Å	Cu–O2/Å	La–O/Å
LM	0	1.941	1.941			2.731
LCM37	1/4	1.945	1.969	1.943	1.970	2.595
LCM55	1/2	1.989	1.934	1.994	1.931	2.599
LCM73	3/4	1.958	1.948	1.957	1.948	2.578
LC	1			2.368	1.883	2.639

a The average bond lengths are given in Å. O1 and O2 refer to the axial and equatorial oxygen atoms of the MO_6_ octahedra, respectively. La–O bond lengths have been included for comparison.

The structural analysis reveals distinct differences in the octahedral distortions among the LCM compounds. Specifically, LCM37 shows a slight compression of both the CuO_6_ and MnO_6_ octahedra. In contrast, LCM55 and LCM73 exhibit a slight octahedral elongation.

In the LC compound, there is a significant distortion of the CuO_6_ octahedra, which should contribute to a more stable AFM ground state.^82,83^ Among the LCM compounds, LCM55 shows the largest deviation between the axial and equatorial bond lengths of both octahedra.

Furthermore, the average La–O bond lengths across these compounds range from 2.578 to 2.731 Å, indicating some variation in the local La environment.

### 3.4 Electronic Structure and Magnetic Properties

To identify the unpaired electrons of the transition metals, which are essential for determining spin states (FM/AFM) in quantum chemical calculations, formal charge analysis was conducted for each compound. This analysis is detailed in Section S6.

The spin states of LM, LC, and LCM perovskites have been calculated based on the formal oxidation states of the atomic species, as presented in Table S7. The primary contributors to the total spin of the system are Mn and Cu atoms, while La and O atoms contribute insignificantly, as confirmed by Mulliken population analysis (Section S7). The calculated unit cells of pristine LM and LC contain two Mn and Cu atoms, respectively. In contrast, the orthorhombic unit cell of the LCM systems contains four Mn sites. For each system, both lower and higher spin configurations have been computed to determine the energetically most favorable spin state. The higher spin configurations of 8, 4, and 4 have been found to be energetically favorable for the LM, LCM37, and LCM55 systems, respectively. For LCM73, a total spin state of 2 has been identified as energetically favorable. These results align with the magnetic measurements, which show a reduction in the magnetic moment with increasing Cu content.

The Mulliken population analysis demonstrated that the total spin and magnetic moment of the compounds are primarily influenced by the Mn and Cu atoms (Section S7). The magnetic moment is significantly dependent on the ratio of Mn(III) and Mn(IV), while Cu(II) atoms contribute only slightly given their populated 3d orbitals (3d^9^). The Mulliken spin population analysis indicated that the spin density of Cu remains largely unchanged with varying Cu content. As expected from the formal charge analysis, La and O do not significantly contribute to the total spin except in LCM73 (Table S16).

Table 2 includes the energy differences between the AFM and FM ground states for the different compounds. As previously mentioned, for LCM37 and LCM73, two distinct FM configurations have been calculated, with LCM73 showing only a small energy difference.

**Table 2:** Energy Difference of the Different Spin States of the LaCu_*x*_Mn_1–*x*_O_3_ Perovskites for Different Cu Concentrations *x*^a^

	*x*	total spin	Δ*E*/eV
LM	0	0	0.3946
		8	0.0000
LCM37	1/4	2	0.8802
		4	0.0000
LCM55	2/4	0	1.1133
		4	0.0000
LCM73	3/4	2	0.0000
		4	0.0470
LC	1	0	0.0000
		2	0.3824

a The energy difference is given in eV.

The observed magnetic properties could be attributed to the interplay of superexchange and double-exchange interactions between the Mn and Cu ions. In LM, FM should be supported by the double-exchange interaction between Mn(III) and Mn(IV) ions.^31^ This interaction is mediated by the O atoms acting as a bridge between the Mn ions. However, Mulliken population analysis has shown that the Mn ions in LM are in the Mn(III) oxidation state, which is inconsistent with the double-exchange mechanism. Cortés-Gil et al.^43^ have shown that a defect-free LM compound exhibits a FM ground state with only Mn(III) ions present, which is in agreement with the theoretical results. The FM state in LM is associated with an indirect exchange mechanism, where the interaction from rare-earth (La) and transition metal (Mn) ions is mediated by the nonmagnetic anion O.^15,84^ The Cu ions in the LC exhibit antiparallel spins through a possible superexchange mechanism, leading to a net spin of zero, due to the AFM interaction between the Cu(II) ions.^31^ As the Cu doping increases in the LCM series, the Mn content decreases, reducing the number of available Mn–Mn pathways for the possible double exchange and thus reducing the total spin and magnetic moment.^32^

In summary, LC adopts an AFM state, while LM has a total spin of 8 per unit cell, representing the FM state. The LCM compounds exhibit total spins of 4, 4, and 2 for LCM37, LCM55, and LCM73, respectively. These findings agree well with data from the experimental measurements, showing a decreasing magnetic moment with increasing Cu content since the latter reduces the total spin of the system by lowering the Mn content.

### 3.5 Band Structure and Density of States

**Figure 4:**
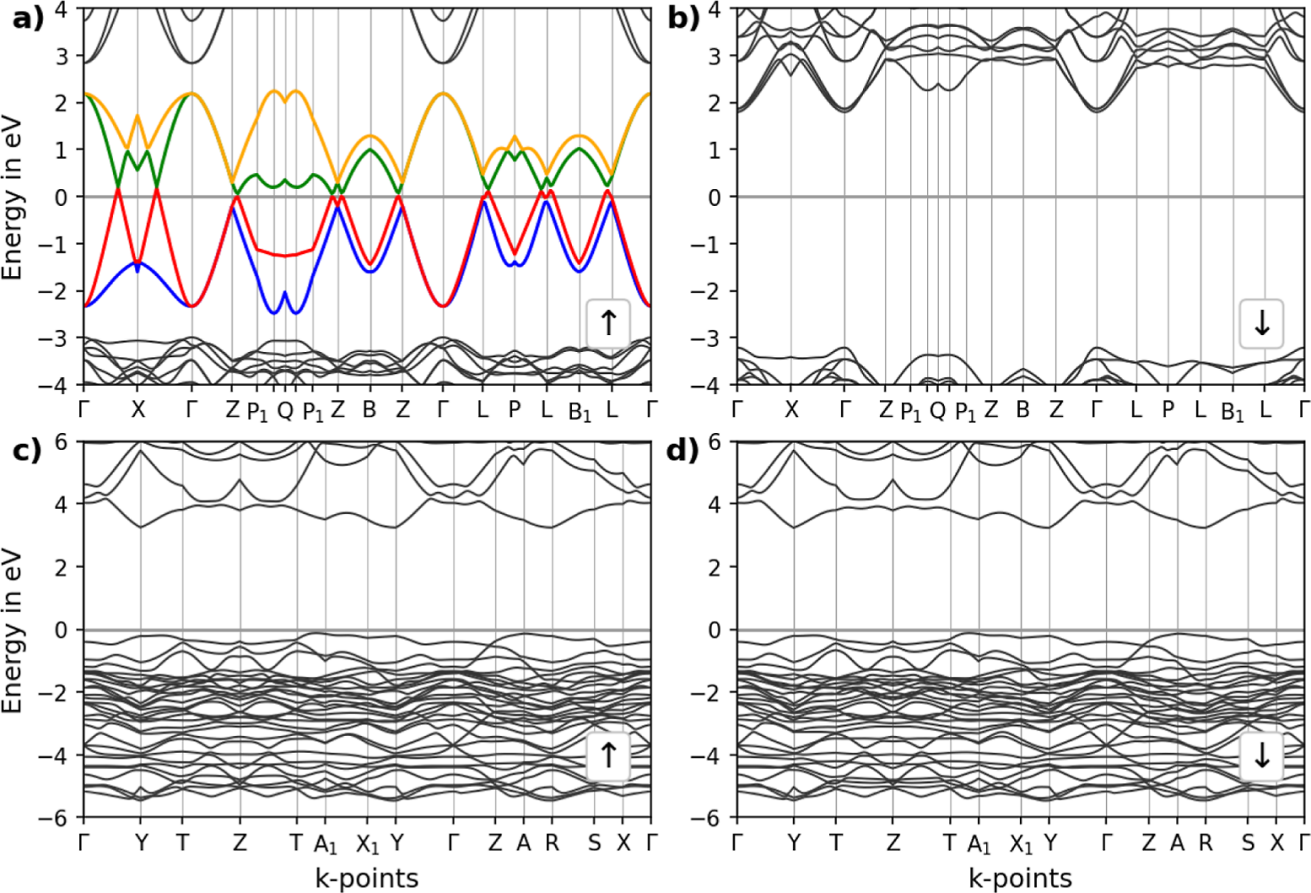
Continuous path band structure of the pristine (a,b) LM and (c,d) LC perovskites. Spin-up and spin-down band structures are shown in (a,c) and (b,d), respectively. To highlight the bands near the Fermi level, different colors are used. The Fermi level is set to 0 eV.

**Figure 5:**
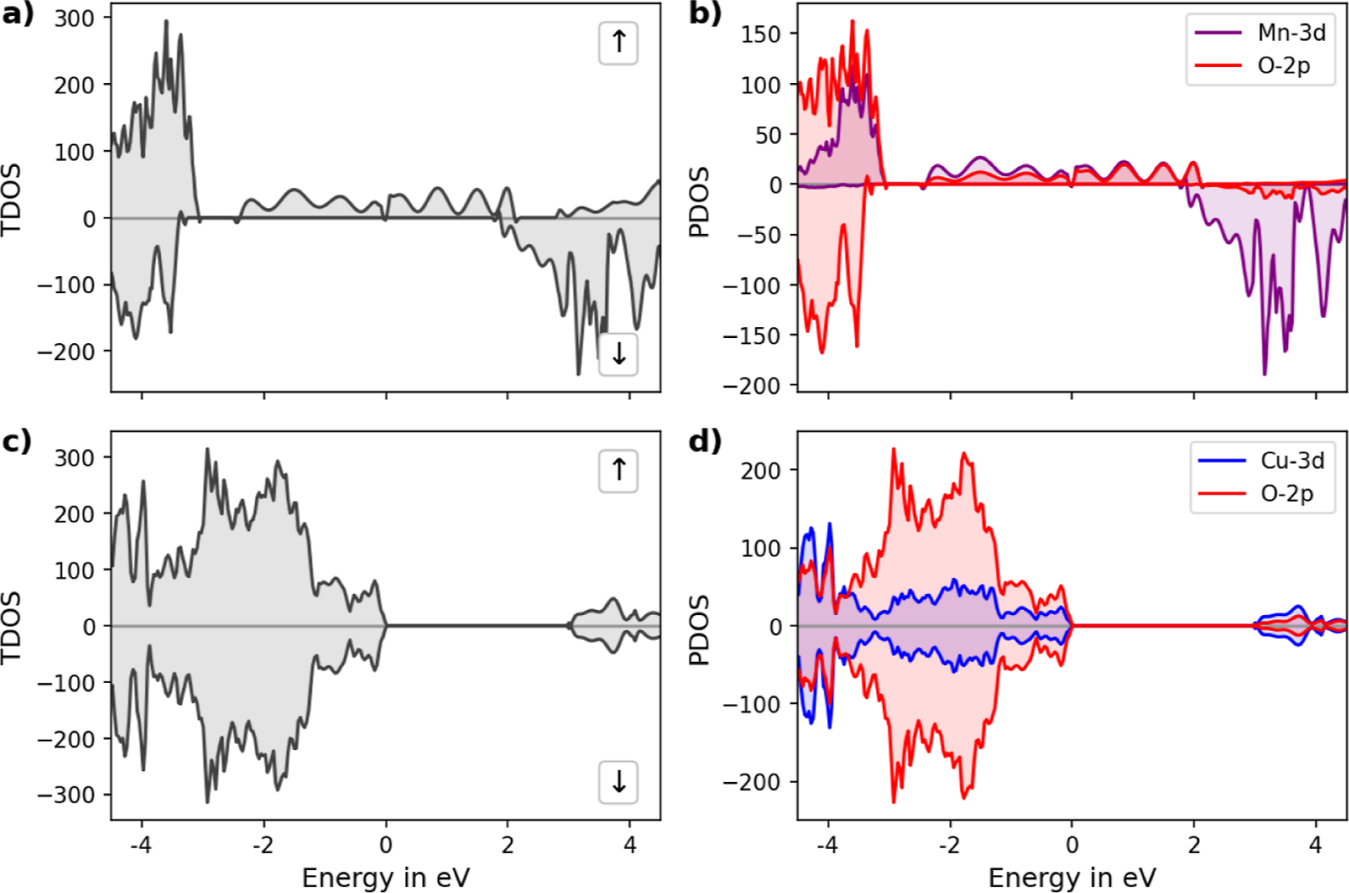
TDOS and PDOS of (a,b) LM and (c,d) LC, respectively. Spin-up and spin-down DOSs are separated by their sign, with spin up having a positive density, while in the spin down case, negative density values are given. The Fermi level is set to 0 eV.

**Figure 6:**
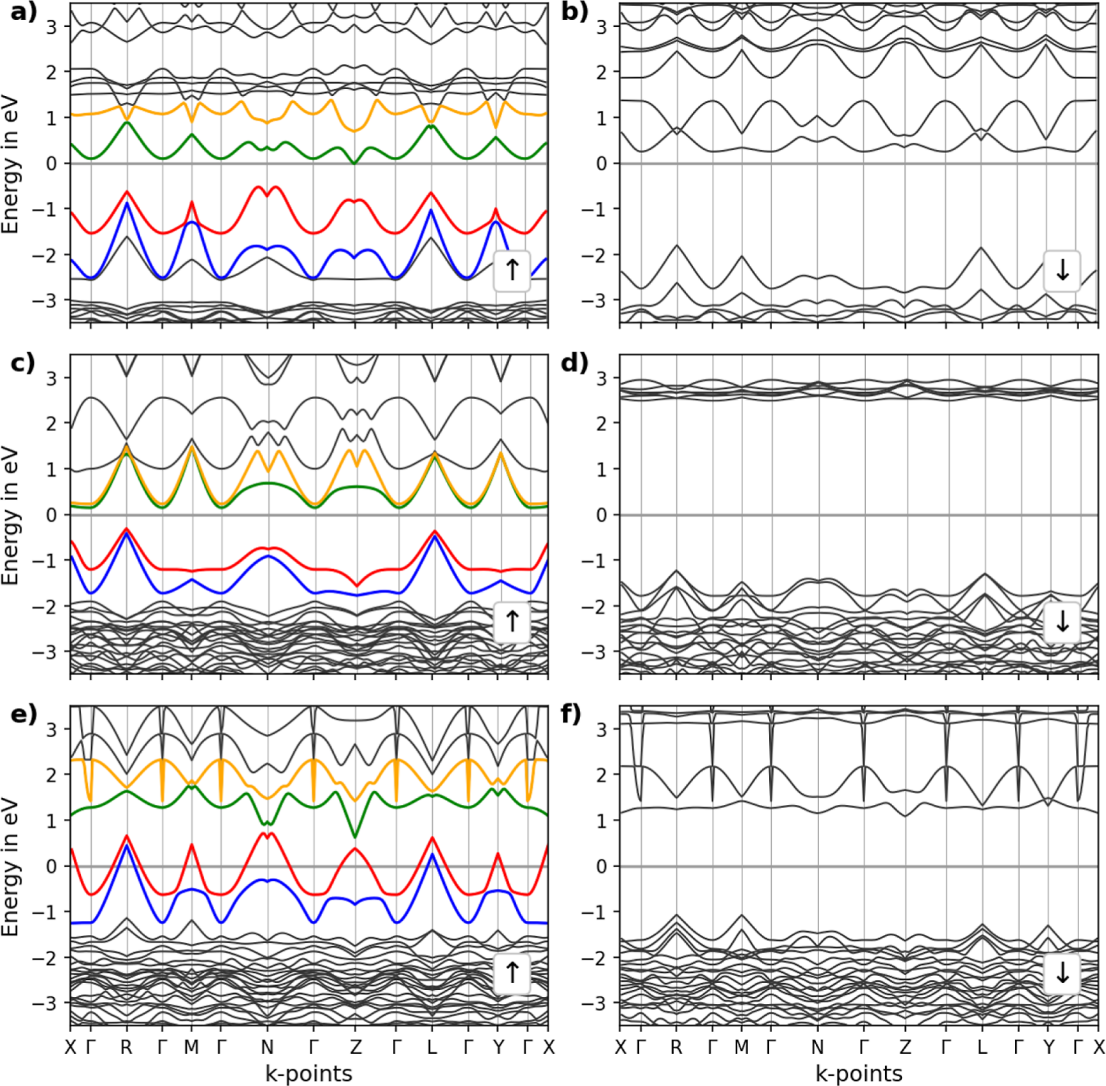
Continuous path band structure of the LCM perovskites. Band structure of (a,b) LCM37, (c,d) LCM55, and (e,f) LCM73. Spin-up and spin-down band structures are shown in (a,c,e) and (b,d,f), respectively. To highlight the bands near the Fermi level, different colors are used. The Fermi level is set to 0 eV.

**Figure 7:**
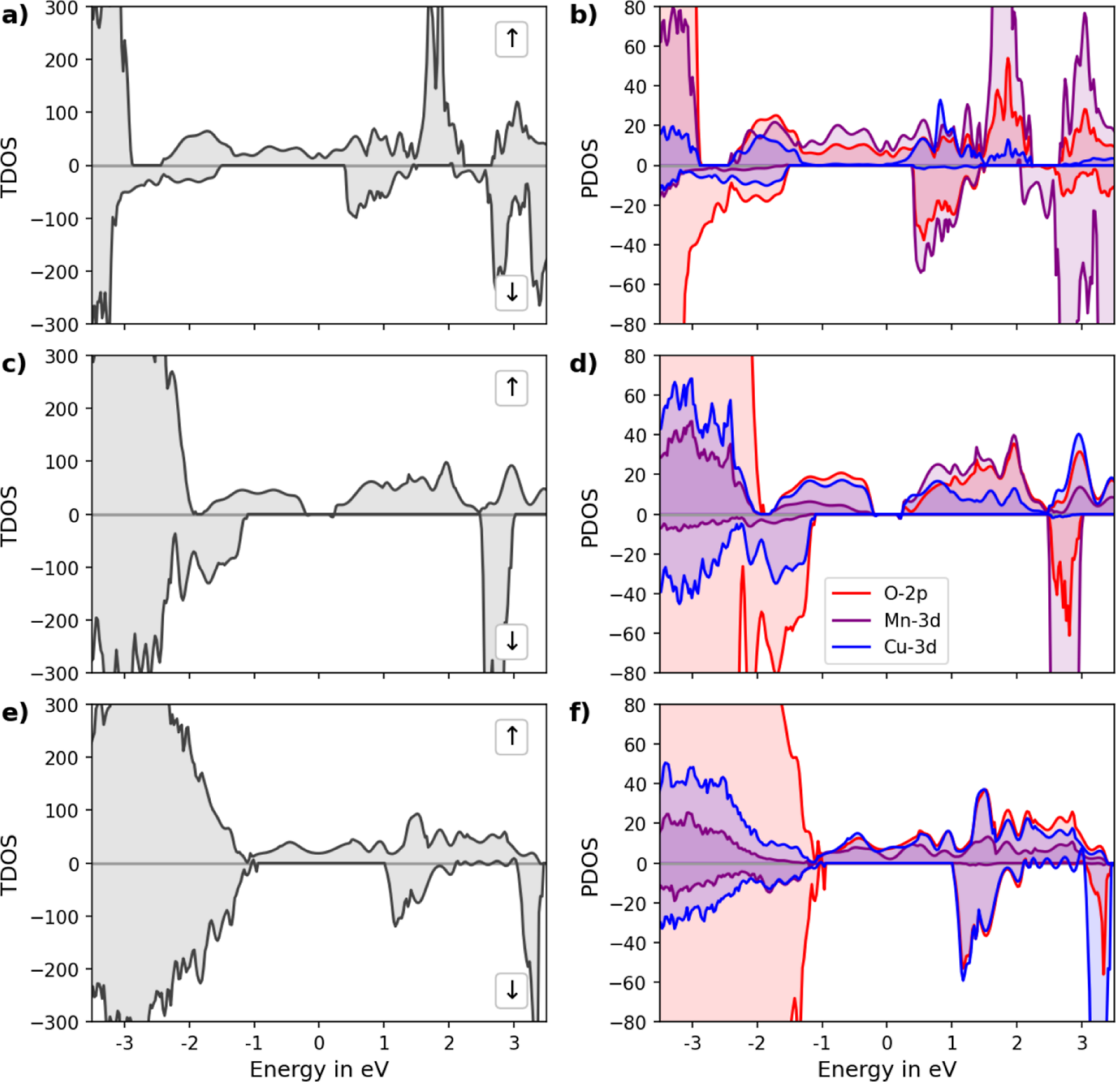
TDOS and PDOS of (a,b) LCM37, (c,d) LCM55, and (e,f) LCM73. Spin-up and spin-down DOSs are separated by their sign, with spin up having positive density values, while spin down is depicted in the negative direction. The Fermi level is set to 0 eV.

The electronic band structures and density of states (DOSs) were calculated to investigate the electronic properties of the pristine and doped perovskites. The continuous path band structures and the associated DOSs of LM and LC perovskites are shown in Figures 4 and 5, while for the doped compounds, the corresponding data are shown in Figures 6 and 7. Additionally, the canonical band structures can be found in Figures S5 and S6. In the case of the doped compounds, the band structure and TDOS/PDOS have been calculated using the transformed crystal structure (*P*1 space group) due to major deviations in the crystal structure after optimization. Therefore, the band path is shown in its *P*1 representation, as can be seen in Figure 3.

For the pristine LM (Figure 4a,b), the spin-up band structure shows substantial half-metallicity with several bands intersecting the Fermi level, indicating 100% spin-polarized electronic conductivity. The band structure clearly indicates the presence of multiple Dirac cones^26,85^ in the path Γ–*X* and on the two high symmetry points *Z* and *L*. The crossing points are slightly above the Fermi level by a value of 0.2 eV. The estimated band gap for LM in the spin-down configuration is approximately 5 eV, while the spin-up configuration shows no band gap.

These observations align well with the findings of Ma et al.,^26^ that have shown a half-metallic behavior of LM based on DFT calculations at the PBE GGA level of theory. Their study highlighted the potential of pristine LaMnO_3_ as a spintronic material due to its unique electronic properties. The presence of Dirac cones and the associated high mobility of spin-polarized electrons make the LM particularly suitable for spintronic applications. Ma et al.^26^ emphasized that the high intrinsic electronic spin polarization in LM could significantly improve the performance and efficiency of spintronic components, positioning this material as a promising candidate for next-generation electronic devices.

LC, in contrast, displays semiconductor characteristics in the spin-up and spin-down configurations, see Figure 4c,d. The AFM ground state inhibits the formation of a spin separation due to the opposed spin states of the Cu atoms in the unit cell. The indirect band gap for LC has been calculated to be approximately 2.9 eV.

Analyzing the TDOS and PDOS provides deeper insights into the orbital contributions within these materials, as shown in Figure 5. For LM, TDOS has confirmed half-metallicity, with PDOS indicating predominant contributions from Mn-3d and O-2p orbitals near the Fermi level. The Mn-3d orbitals show an increased density of the spin-down states after 2 eV, while below approximately −3 eV, the spin-up states show a higher density. LC PDOS shows significant contributions from both the Cu 3d and the 2p orbitals near the Fermi level. TDOS and PDOS of LC show a clear band gap of approximately 2.9 eV, consistent with the band structure calculations.

In the Cu-doped variants, LCM37 features band crossings (*k*-point *Z*) at the Fermi level in the spin-up configuration, again indicating half-metallic behavior. The spin-down counterpart displayed no crossings and different band patterns, suggesting significant spin-dependent electronic states. Although Figure 6a shows a band gap of approximately 0.2 eV for the spin-up configuration, the canonical band structure (Figure S6a) reveals that no band gap is present. Figure S6a shows two band crossings in the path U–R–T and one band crossing in the path U–X. In the paths R–T and S–R, the band structure reveals that the valence band maximum is located above the conduction band minimum, indicating conducting properties. This is consistent with TDOS and PDOS (Figure 7a,b), which show significant state densities around the Fermi level for the spin-up configuration. In contrast, the spin-down configuration shows a larger gap of about 2 eV.

LCM55 exhibits an indirect band gap of around 0.2 eV for spin-up and approximately 3.5 eV for spin-down states. LCM73 does not show a band gap in the spin-up bands, while the spin-down bands show a gap of approximately 2 eV, see Figure 6c–f. The canonical band structure of LCM73 (Figure S6c) shows that the valence band maximum is located above the conduction band minimum, indicating 100% spin-polarized conducting properties. Additionally, several band crossings are present. The band structure shows Dirac cones at the *k*-points *L* and *Z* with a Dirac gap of about 0.2 eV (see Figure 6c,d).

This data clearly shows that the half-metallic properties of the LaMnO_3_ lead structure can be effectively steered via Cu doping, which proves particularly interesting for the engineering of spintronic materials.

For the Cu-doped perovskites, the TDOS in LCM37, LCM55, and LCM73 shows significant state densities around the Fermi level for the spin-up configurations. The PDOS has highlighted the dominant roles of Mn 3d and Cu 3d orbitals alongside the contributions of the 2p and the 3d orbitals, which are crucial for understanding the electronic interactions and the impact of Cu doping within these complex oxides. In LCM37, the PDOS shows a significant contribution from the Mn-3d and O-2p orbitals, with a relatively small density from the Cu-3d orbitals at the Fermi level. At higher energies, the Cu 3d orbitals show an increase in the number of states. In LCM55, PDOS shows a similar trend, with a slight increase in the Cu-3d orbital contribution below the Fermi level. Below the Fermi level, the Mn-3d orbitals show a decrease in density, while above the Fermi level, the density has increased significantly. The band gap opening in LCM55 has been attributed to the JT *z* elongation of the CuO_6_ and MnO_6_ octahedra, see Table 1. This axial elongation breaks the symmetry of the system, leading to a band gap opening. In LCM55, the PDOS shows a substantial contribution of all significant orbitals at the Fermi level, although the contribution of the Mn-3d orbitals is decreasing at the Fermi level, whereas the Cu-3d and O-2d orbitals’ density remains relatively constant.

## 4 Conclusions

Analysis of copper-doped lanthanum manganite (LaCu_*x*_Mn_1–*x*_O_3_) perovskites has provided a detailed understanding of how Cu incorporation influences the structural, electronic, and magnetic properties of these materials.

Magnetic measurements reveal a clear dependence of the Curie temperature on the Cu content with a notable decrease in *T*_C_ as the Cu concentration increases. This trend suggests that copper doping alters the magnetic interactions within the material, likely by modifying the Mn(III)/Mn(IV) ratio. It has been shown that the Cu-doped perovskites only decrease the presence of Mn(III)/Mn(IV) ions, and the presence of Cu(II) ions does not significantly contribute to the magnetic moment. The observed FM behavior at low temperatures and the reduction in magnetic moment with higher Cu content align well with the theoretical predictions and magnetic resonance measurements.

Theoretical structural optimization of the doped compounds has revealed that the mixed Mn/Cu perovskites exhibit a suppressed JT distortion. The band structure and DOS analysis indicate that the copper doping affects the half-metallicity of LaMnO_3_, with a significant difference in the spin-down band gap. The robust electronic spin polarization, especially upon varying levels of Cu doping, underscores these materials’ potential in spintronic applications, where spin-dependent transport properties are crucial.^86^ Additionally, their potential use in catalysis is enhanced by the ability to fine-tune the electronic structure. The half-metallicity observed in the samples suggests their suitability for applications requiring high electrical conductivity. Furthermore, the intricate orbital interplay near the Fermi level points to potential uses in catalytic and electrocatalytic applications, particularly in reactions related to energy conversion and storage technologies.^87^

Overall, this study highlights the potential of Cu-doped LaMnO_3_ perovskites in advanced technological applications, driven by their customizable structural, magnetic, and electronic properties. The insights gained from this research pave the way for further exploration and optimization of perovskite doped materials for a broad range of functional applications.

## Data Availability

The scripts used for the high symmetry path generation have been deposited on Zenodo and can be accessed via the following link https://zenodo.org/doi/10.5281/zenodo.10727586. The magnetic and cw-EPR measurements data are available via the following link https://zenodo.org/doi/10.5281/zenodo.13690823.

## Supporting Information Available

Experimental details for ICP, BET, XPS, and XRD analyses; PXRD data for various samples; optimization results for lattice configurations; lattice parameter measurements; magnetization and cw-EPR measurements; theoretical oxidation and spin states; Mulliken population analysis; and electronic band structure representations (PDF)
