# Supplementary material for: Combined Experimental and Theoretical Approach to the Electronic and Magnetic Properties of Cu-Doped LaMnO3 Perovskites
Source: J Phys Chem C Nanomater Interfaces. 2024 Dec 23;129(1):677–88. doi: 10.1021/acs.jpcc.4c06256 (PMC11726620; doi:10.1021/acs.jpcc.4c06256)
Supplement: Supplementary file 1 — jp4c06256_si_001.pdf [file jp4c06256_si_001.pdf]

Supporting Information:

A combined experimental and theoretical approach to the electronic and magnetic properties of Cu-doped $LaMnO_3$ perovskites

Josef M. Gallmetzer [†], Felix R. S. Purtscher [†], Jakob Gamper [†], Mohammadi Asghar [‡], Ralf Feyerherm [¶], Wiebke Riedel [§], Simon Penner [‡], and Thomas S. Hofer [*,†]

†*Institute of General, Inorganic and Theoretical Chemistry*
University of Innsbruck, Innrain 80-82,
6020 Innsbruck, Austria

‡*Institute of Physical Chemistry*
University of Innsbruck, Innrain 52c,
6020 Innsbruck, Austria

¶*Institute Quantum Phenomena in Novel Materials,*
Helmholtz-Zentrum Berlin für Materialien und Energie GmbH, Hahn-Meitner-Platz 1,
14109 Berlin, Germany

§*Institute of Physical and Theoretical Chemistry*
Free University of Berlin, Arnimallee 22,
14195 Berlin, Germany

E-mail: T.Hofer@uibk.ac.at

Phone: +43-512-507-57111. Fax: +43-512-507-57199

S1 Experimental Details for ICP, BET, XPS and XRD

Elemental analysis for lanthanum (La), copper (Cu) and manganese (Mn) were performed with inductively coupled plasma optical emission spectroscopy (ICP-OES) by using a Horiba Scientific ICP Ultima2 (Horiba, Kyoto, Japan). Prior to analysis, powder samples were dissolved in an aqueous suspension of HNO_3 and HF acid mixture at 200 °C for 5 h in a Teflon-lined autoclave. BET-based specific surface areas were measured by means of nitrogen sorption, using a Quanta- Chrome Nova 2000e surface and pore size analyzer and liquid N_2 as adsorbent. Before testing, the samples were degassed at 270 °C in vacuo for 1 h.

Powder X-ray diffraction patterns of the initial samples were recorded with a Rigaku SmartLab-SE diffractometer in focusing beam setting and reflections mode on a Θ/Θ goniometer and Co-Kα radiation, ($\lambda = 1.78900$ Å) using a D/teX Ultra 250 compound silicon strip 1D-detector. Patterns were recorded with an incident slit of 0.5° in a range from 5° to 80° 2Θ with a step width of 0.005° and a speed of 0.5° min^{-1}. The software SmartLab Studio-II (Rigaku Corporation, 2014) was used for diffractometer control and phase assignment using the PDF-4+ (International Centre for Diffraction Data 2023) database. Rietveld analysis was performed using the HighScore Plus software. Peaks were fitted by functions with corresponding Gauss and Lorentz parts.

X-ray photoelectron spectroscopy (XPS) measurements were performed using a VG ESCALAB apparatus and a monochromated Al-K_α X-ray source (E = 1487 eV). XP spectra were collected after initial calcination of the samples. Evaluation of the data was performed using the CasaXPS software. For calculation of the surface ion concentration, relative sensitivity factor (RSF) and electron mean free path corrections have been applied. Detailed information is available in Ref.[S1]

Table S1: Overview of nominal bulk catalyst composition, BET-derived specific surface area (SSA), bulk composition ICP and surface composition XPS analysis.

Nominal Composition	Acronyms	SSA / m^2g^{-1}	Chemical composition based on ICP	Chemical composition based on XPS
$LaMnO_3$	LM	16.6	$La_{1.03}Mn_{0.97}O_{3-\delta}$	-
$LaCu_{0.3}Mn_{0.7}O_3$	LCM37	21.5	$La_{1.07}Cu_{0.29}Mn_{0.64}O_{3-\delta}$	$La_{1.65}Cu_{0.09}Mn_{0.26}O_{3-\delta}$
$LaCu_{0.5}Mn_{0.5}O_3$	LCM55	13.6	$La_{1.07}Cu_{0.49}Mn_{0.44}O_{3-\delta}$	$La_{1.57}Cu_{0.31}Mn_{0.12}O_{3-\delta}$
$LaCu_{0.7}Mn_{0.3}O_3$	LCM73	15.8	$La_{1.04}Cu_{0.69}Mn_{0.26}O_{3-\delta}$	$La_{1.60}Cu_{0.33}Mn_{0.08}O_{3-\delta}$
La_2CuO_4	LC	12.0	$La_{1.04}Cu_{0.96}O_{3-\delta}$	-

S2 Powder X-ray Diffraction (PXRD)

The LC system consists of three phases, La_2CuO_4, CuO and $La_2O_2CO_3$, which are fully compatible with the following reference structures. The LCM systems LCM37 and LCM55 consist of a single phase of $LaCu_xMn_{1-x}O_3$, which matches the reference pattern of $La_{0.996}Cu_{0.4}Mn_{0.6}O_3$. The LCM73 system matches the $La_{0.996}Cu_{0.4}Mn_{0.6}O_3$ pattern and, in addition, impurities consisting of La_2CuO_4 and CuO have been identified.

- $La_2CuO_{4.09}$:[S2]
  - Crystal system: orthorhombic
  - Space group: *Fmmm* (69)
  - ICSD coll. Code: 41494
  - Cell Parameters: 5.34(2), 5.34(2), 13.23(3), 90. 90. 90.
- CuO:[S3]
  - Crystal system: monoclinic
  - Space group: *C12/c1* (15)
  - ICSD coll. Code: 16025
  - Cell Parameters: 4.6837(5), 3.4226(5), 5.1288(6), 90. 99.54(1) 90.
- $La_2O_2CO_3$:[S4]
  - Crystal system: hexagonal
  - Space group: $P6_3/mmc$ (194)
  - ICSD coll. Code: 202988
  - Cell Parameters: 4.0755(6), 4.0755(6), 15.957(1), 90. 90. 120.
- $LaMnO_3$:[S5]
  - Crystal system: trigonal
  - Space group: $R\overline{3}c$ (167)
  - ICSD coll. Code: 37317
  - Cell Parameters: 5.51053(18), 5.51053(18), 13.39558(74), 90. 90. 120.

- $La_{0.996}Cu_{0.4}Mn_{0.6}O_3$:[S6]
  - Crystal system: orthorhombic
  - Space group: *Pbnm* (62)
  - ICSD coll. Code: 29193
  - Cell Parameters: 5.5257(3), 5.4904(3), 7.7744(5), 90. 90. 90.

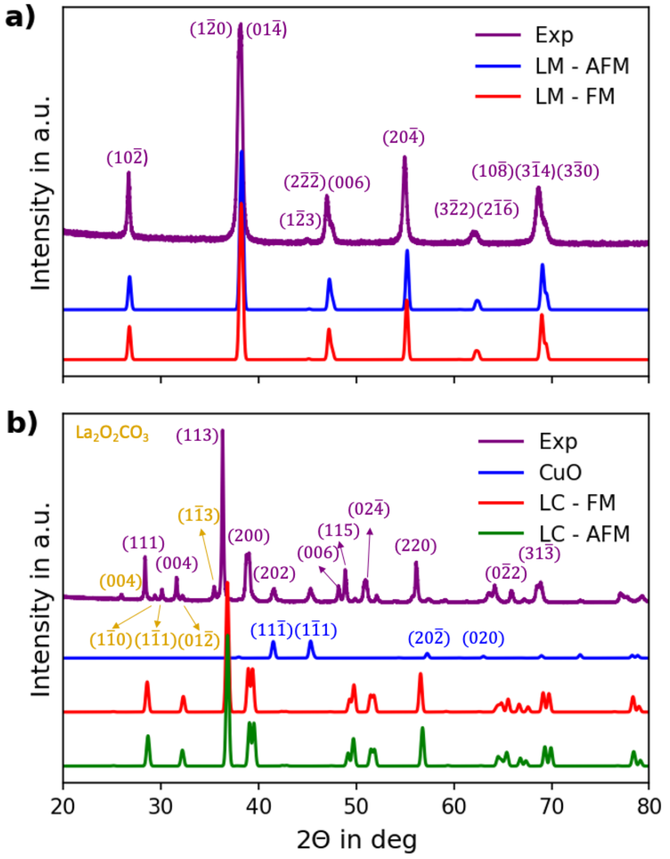

Figure S1: Powder X-ray diffraction pattern of a) LM and b) LC perovskites. The intensities are normalized to the highest reflex. b) CuO impurities are clearly visible in the La_2CuO_4 (LC) sample, indicated by the PXRD pattern of CuO.[S7] The wavelength of the X-ray radiation corresponds to the Co-Kα line ($\lambda = 1.79026$ Å). Miller indices are given for all reflexes.

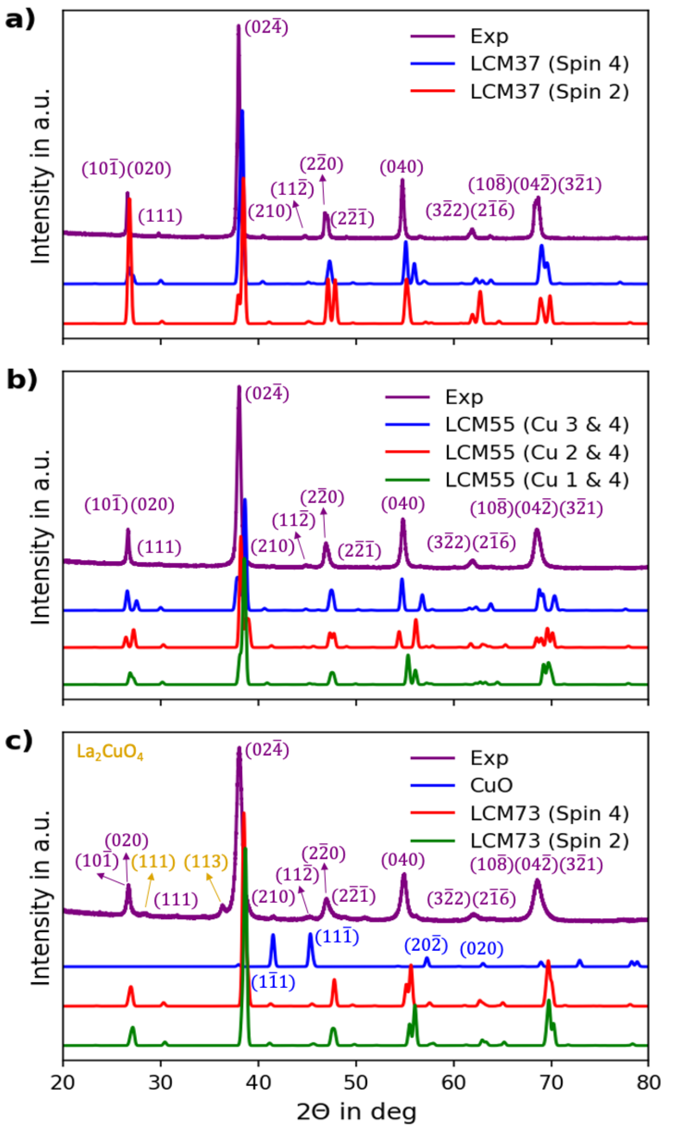

Figure S2: Powder X-ray diffraction pattern of La(Mn,Cu)O_3 perovskites. a) LCM37, b) LCM55 and c) LCM73. The intensities are normalized to the highest reflex. c) Contains reference CuO[S7] theoretical calculated PXRD pattern. The wavelength of the X-ray radiation corresponds to the Co-Kα line ($\lambda = 1.79026$ Å). Miller indices are given for all reflexes.

S3 Optimization Results - Configurations LCM55

Table S2: Total energy differences of the LCM55 configuration isomers with respect to the lowest energy configuration, *i.e.* Cu substitution being at the Mn/Cu lattice sites S_2 and S_4.

Position	S_1	S_2	S_3	S_4	ΔE / eV
14	Cu	Mn	Mn	Cu	0.3613
24	Mn	Cu	Mn	Cu	0.0000
34	Mn	Mn	Cu	Cu	0.8585

S4 Lattice parameters of LCM, LM and LC

Table S3: Lattice parameters of LCM perovskites in the orthorhombic structure (space group no. 62; *Pnma*)[S6] after optimization.

	a /Å	b /Å	c /Å	α /°	β /°	γ /°
Exp	5.526	7.774	5.490	90.000	90.000	90.000
LCM37	5.492	7.623	5.475	89.867	90.302	89.691
LCM55	5.357	7.824	5.396	89.604	90.000	90.000
LCM73	5.397	7.684	5.444	90.116	90.737	90.066

Table S4: Lattice parameters of $LaMnO_3$ (LM) in the rhombohedral structure (space group no. 167; $R\bar{3}c$)[S5] after optimization.

	a /Å	b /Å	c /Å	α /°	β /°	γ /°
Exp	5.510	5.510	13.396	90	90	120
Calc	5.521	5.521	13.209	90	90	120

Table S5: Lattice parameters of La_2CuO_4 (LC) in the orthorhombic structure (space group no. 64; *Bmab*)[S8] after optimization.

	a /Å	b /Å	c /Å	α /°	β /°	γ /°
Exp	5.352	5.4	13.157	90	90	90
Calc	5.290	5.350	12.900	90	90	90

S5 Magnetic Measurements

S5.1 Magnetization Measurements

The magnetization measurements show with increasing Cu-content a decrease in magnetization at high magnetic fields for both low temperature and room temperature (see Figure S3). The effective magnetic moment determined for the low temperature measurements decreases accordingly (LM: 4.8; LCM37: 4.6; LCM55: 3.6 and LCM73: 2.3) which agrees roughly with a previous study on similar samples.[S9] The coercivity at room temperature is overall small, as expected for systems above the ferromagnetic transition temperature. The samples with a higher Cu-content (LCM55, LCM73 and LC), a small, but notable coercivity (150-200) Oe presumably due to a small ferromagnetic impurity (coercivities $\leq$ 50 Oe are rather experimental artifacts). At low temperatures, the coercivity initially increases with increasing Cu-content, but decreases as the Cu-content is further raised. This attests to Cu affecting the magnetic system, but also indicates that its effects are complex.

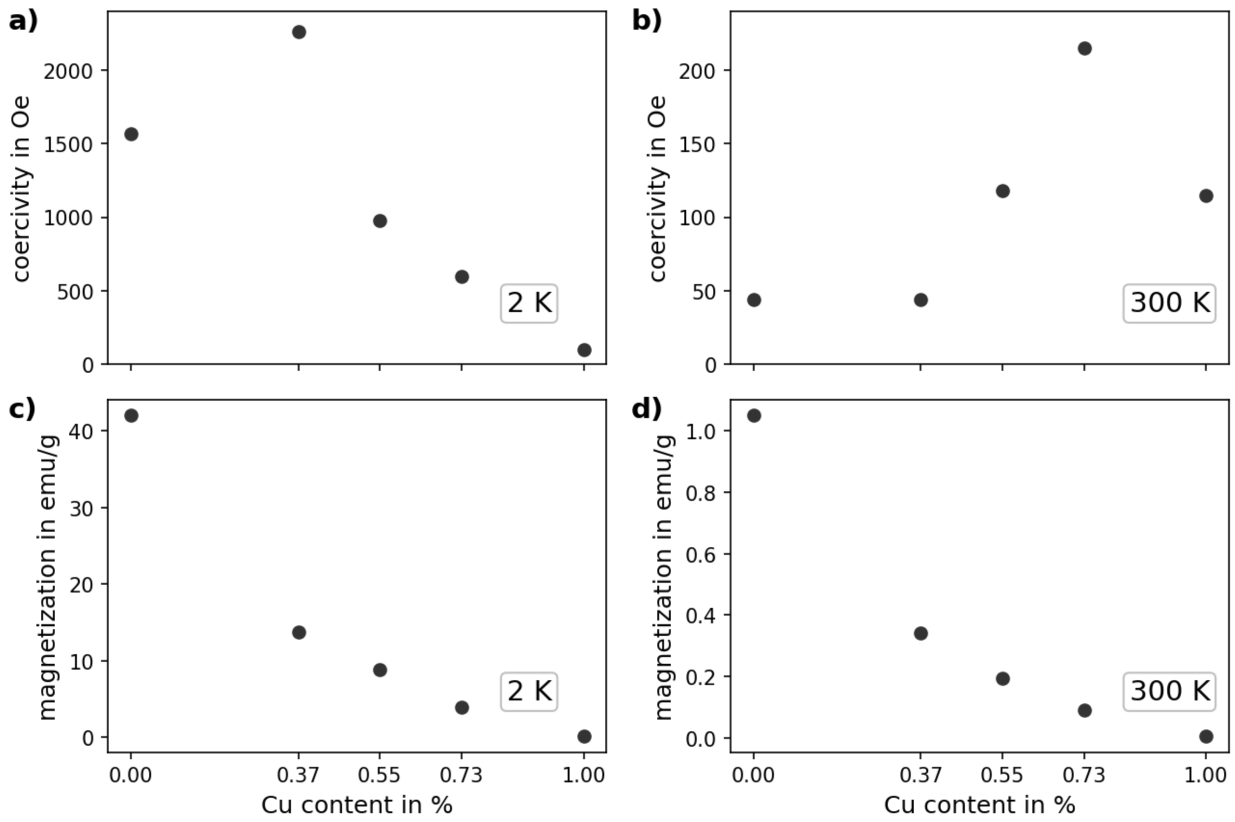

Figure S3: a) Coercivity and c) magnetic moment at 5 T (50 000 Oe) for the 2 K measurements as a function of the Cu-content x. b) Measured coercivity as a function of Cu-content x. d) Magnetic moment at 1 T (10 000 Oe) as a function of the Cu-content.

S5.2 cw-EPR Measurements

The cw-EPR measurements conducted at room temperature exhibit for all LCM samples as well as the LM reference a broad signal around $g_{eff} = 2$ consistent with a strongly coupled spin-system. In contrast, the LC references does not show notable signals in the cw EPR spectra, in agreement with an antiferromagnetic system with a Néel-temperature $> 300\,K$. With increasing Cu-content, the amplitude and (double-integrated) intensity of the signals decreases (see Figure S4). This agrees both with the magnetization measurements as well as with a previous study on similar samples.[S9] The g_{eff} increases from 1.99 to 2.02. The ΔB_{pp} linewidth varies with the Cu-content, but no clear trend is observed. Thus, the presence of Cu clearly affects the strongly coupled spin-system, but the influence on the cw-EPR signals is complex and depends on its concentration.

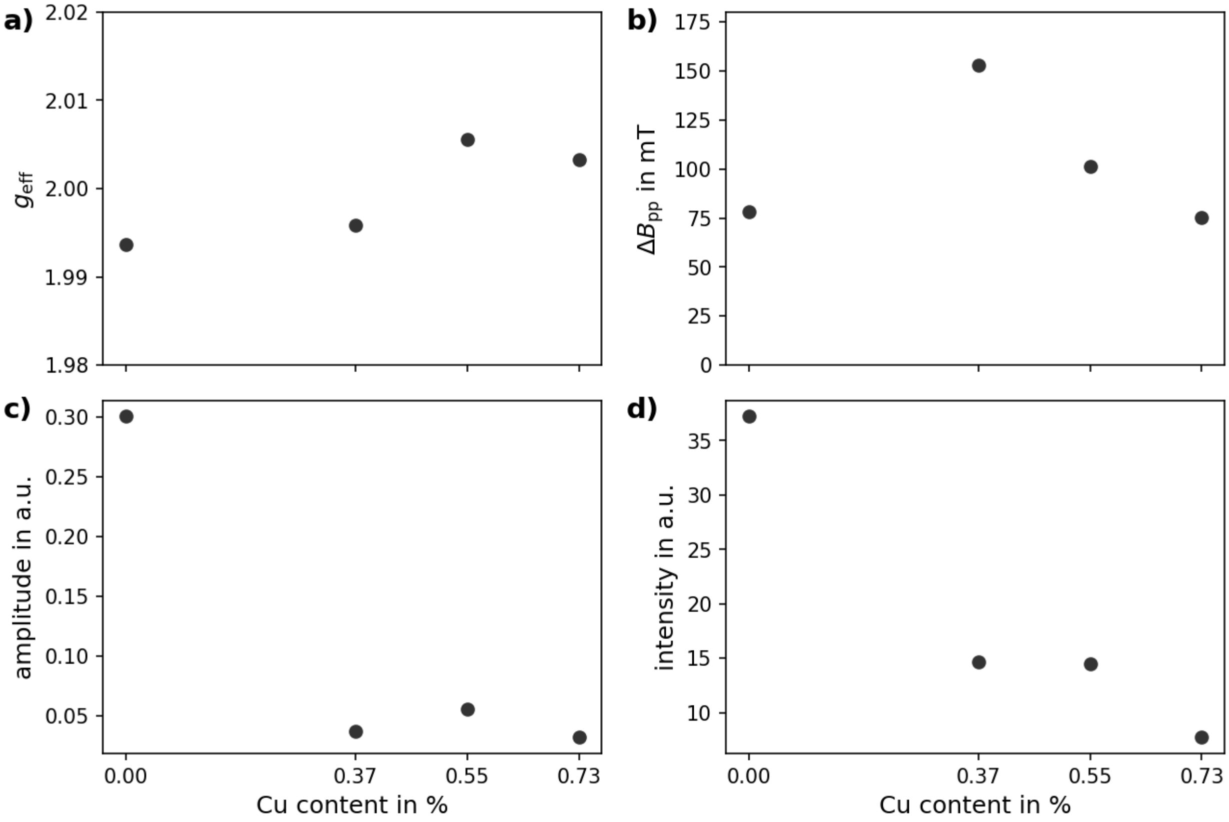

Figure S4: Effective g-value g_{eff} (a), line width B_{pp} (b), amplitude (peak to peak) (c) and (double-integrated) intensity (d) of cw-EPR measurements conducted at room temperature for LM and LCM samples as a function of the Cu-content.

S6 Theoretical Oxidation and Spin States

The oxidation states of the atoms have been analyzed by taking the formal charges (oxidation number) of the anionic and cationic species into account. In particular, the Mn cations are known to exhibit a high degree of variability in their oxidation states. Consequently, as the Cu content increases, the oxidation state of Mn has been set from +III, in the pristine compound LM, ranging up to +VI, in the compound with the highest Cu content (LCM73) in order to ensure charge neutrality of the chemical system, see Table S6. To ensure charge neutrality of the LCM73 system, Mn is in its formal +VI oxidation state. Mulliken population analysis showed that the oxidation state of Mn is formally +III or +IV in nearly all cases, see Section S7.

Table S6: Oxidation states of $LaCu_xMn_{1-x}O_3$ of the formal charge analysis. $x = 1$ corresponds to La_2CuO_4 and $x = 0$ to $LaMnO_3$. To achieve formally neutral compounds, the oxidation state of Mn, in the case of $x = 3/4$, is given as +VI, although this is not common. Therefore, the oxidation state of (+VI) is given in brackets. The Mulliken analysis later showed that the oxidation state of Mn is formally +IV.

	x	La	Cu	Mn	O
LM	0	+III	-	+III	-II
LCM37	1/4	+III	+II	+III / +IV	-II
LCM55	1/2	+III	+II	+IV	-II
LCM73	3/4	+III	+II	(+VI)	-II
LC	1	+III	+II	-	-II

Table S7: Number of unpaired electrons and the respective spin orientation given as the sign of $LaCu_xMn_{1-x}O_3$ perovskites for different Cu content x. The total spin is calculated as the sum of the magnetic moments at the Mn/Cu sites X. The spin of the electrons is given in $2 \cdot m_S$.

	x	X_1	X_2	X_3	X_4	$2 \cdot$ Total Spin
LM	0	+4	+4	-	-	0
		+4	+4	-	-	8
LCM37	1/4	−1	+3	+4	−4	2
		−1	−3	+4	+4	4
LCM55	2/4	+3	+1	−3	−1	0
		+3	−1	+3	−1	4
LCM73	3/4	−1	+1	−1	+3	2
		+1	+1	−1	+3	4
LC	1	+1	−1	-	-	0
		+1	+1	-	-	2

S7 Mulliken Population Analysis

S7.1 Mulliken Population Analysis - Charge

Table S8: Mulliken charge population analysis of $LaMnO_3$ (LM) after optimization. The total number of electrons regarding the La atoms is given in brackets due to the employed ECP replacing 46 core electrons.

Atom	Element	Atom no.	Occupation	Atom Charge / e
1	La	57 (11)	9.174	+1.826
2	La	57 (11)	9.174	+1.826
3	Mn	25	23.686	+1.314
4	Mn	25	23.686	+1.314
5	O	8	9.047	−1.047
6	O	8	9.047	−1.047
7	O	8	9.047	−1.047
8	O	8	9.047	−1.047
9	O	8	9.047	−1.047
10	O	8	9.047	−1.047

Table S9: Mulliken charge population analysis of LCM37 after optimization. The total number of electrons regarding the La atoms is given in brackets due to the employed ECP replacing 46 core electrons.

Atom	Element	Atom no.	Occupation	Atom Charge / e
1	Cu	29	27.929	+1.071
2	Mn	25	23.651	+1.349
3	Mn	25	23.674	+1.326
4	Mn	25	23.659	+1.341
5	La	57 (11)	9.149	+1.851
6	La	57 (11)	9.152	+1.848
7	La	57 (11)	9.152	+1.848
8	La	57 (11)	9.149	+1.851
9	O	8	9.036	−1.036
10	O	8	9.030	−1.030
11	O	8	9.030	−1.030
12	O	8	9.036	−1.036
13	O	8	9.038	−1.038
14	O	8	9.021	−1.021
15	O	8	9.056	−1.056
16	O	8	9.063	−1.063
17	O	8	9.038	−1.038
18	O	8	9.021	−1.021
19	O	8	9.056	−1.056
20	O	8	9.063	−1.063

Table S10: Mulliken charge population analysis of LCM55 after optimization. The total number of electrons regarding the La atoms is given in brackets due to the employed ECP replacing 46 core electrons.

Atom	Element	Atom no.	Occupation	Atom Charge / e
1	Mn	25	23.639	+1.361
2	Cu	29	27.956	+1.044
3	Mn	25	23.639	+1.361
4	Cu	29	27.956	+1.044
5	La	57 (11)	9.136	+1.864
6	La	57 (11)	9.136	+1.864
7	La	57 (11)	9.136	+1.864
8	La	57 (11)	9.136	+1.864
9	O	8	9.028	−1.082
10	O	8	9.028	−1.082
11	O	8	9.028	−1.082
12	O	8	9.028	−1.082
13	O	8	9.018	−1.018
14	O	8	9.019	−1.019
15	O	8	9.018	−1.018
16	O	8	9.019	−1.019
17	O	8	9.018	−1.018
18	O	8	9.019	−1.019
19	O	8	9.018	−1.018
20	O	8	9.019	−1.019

Table S11: Mulliken charge population analysis of LCM73 after optimization. The total number of electrons regarding the La atoms is given in brackets due to the employed ECP replacing 46 core electrons.

Atom	Element	Atom no.	Occupation	Atom Charge / e
1	Cu	29	27.922	+1.078
2	Cu	29	27.897	+1.103
3	Cu	29	27.936	+1.064
4	Mn	25	23.655	+1.345
5	La	57 (11)	9.138	+1.862
6	La	57 (11)	9.134	+1.866
7	La	57 (11)	9.134	+1.866
8	La	57 (11)	9.138	+1.862
9	O	8	9.004	−1.004
10	O	8	8.998	−0.998
11	O	8	8.998	−0.998
12	O	8	9.004	−1.004
13	O	8	8.999	−0.999
14	O	8	8.992	−0.992
15	O	8	9.012	−1.012
16	O	8	9.017	−1.017
17	O	8	8.999	−0.999
18	O	8	8.992	−0.992
19	O	8	9.012	−1.012
20	O	8	9.017	−1.017

Table S12: Mulliken charge population analysis of La_2CuO_4 (LC) after optimization. The total number of electrons regarding the La atoms is given in brackets due to the employed ECP replacing 46 core electrons.

Atom	Element	Atom no.	Occupation	Atom Charge / e
1	La	57 (11)	9.346	+1.654
2	La	57 (11)	9.346	+1.654
3	La	57 (11)	9.346	+1.654
4	La	57 (11)	9.346	+1.654
5	Cu	29	28.040	+0.960
6	Cu	29	28.040	+0.960
7	O	8	9.056	−1.056
8	O	8	9.056	−1.056
9	O	8	9.056	−1.056
10	O	8	9.056	−1.056
11	O	8	9.077	−1.077
12	O	8	9.077	−1.077
13	O	8	9.077	−1.077
14	O	8	9.077	−1.077

S7.2 Mulliken Population Analysis - Spin

Table S13: Mulliken spin population analysis of LM after optimization.

Atom	Element	$2 \cdot$ Spin / -
1	La	+0.001
2	La	+0.001
3	Mn	+4.087
4	Mn	+4.087
5	O	−0.029
6	O	−0.029
7	O	−0.029
8	O	−0.029
9	O	−0.029
10	O	−0.029
Sum		8.000

Table S14: Mulliken spin population analysis of LCM37 after optimization.

Atom	Element	$2 \cdot$ Spin / -
1	Cu	−0.723
2	Mn	−3.132
3	Mn	+4.133
4	Mn	+3.855
5	La	+0.003
6	La	+0.002
7	La	+0.002
8	La	+0.003
9	O	−0.045
10	O	+0.025
11	O	+0.025
12	O	−0.045
13	O	−0.045
14	O	+0.025
15	O	−0.020
16	O	−0.012
17	O	−0.045
18	O	+0.025
19	O	−0.020
20	O	−0.012
Sum		4.000

Table S15: Mulliken spin population analysis of LCM55 after optimization.

Atom	Element	2 · Spin / -
1	Mn	+3.236
2	Cu	−0.747
3	Mn	+3.236
4	Cu	−0.747
5	La	+0.011
6	La	+0.011
7	La	+0.011
8	La	+0.011
9	O	−0.075
10	O	−0.075
11	O	−0.075
12	O	−0.075
13	O	−0.098
14	O	−0.083
15	O	−0.098
16	O	−0.083
17	O	−0.098
18	O	−0.083
19	O	−0.098
20	O	−0.083
Sum		4.000

Table S16: Mulliken spin population analysis of LCM73 after optimization.

Atom	Element	$2 \cdot$ Spin / -
1	Cu	−0.786
2	Cu	+0.825
3	Cu	−0.778
4	Mn	+3.477
5	La	+0.008
6	La	+0.009
7	La	+0.009
8	La	+0.008
9	O	−0.156
10	O	+0.020
11	O	+0.020
12	O	−0.156
13	O	+0.012
14	O	+0.008
15	O	−0.128
16	O	−0.142
17	O	+0.012
18	O	+0.008
19	O	−0.128
20	O	−0.142
Sum		2.000

Table S17: Mulliken spin population analysis of LC after optimization.

Atom	Element	$2 \cdot$ Spin / -
1	La	+0.001
2	La	−0.001
3	La	+0.001
4	La	−0.001
5	Cu	−0.601
6	Cu	+0.601
7	O	−0.004
8	O	+0.004
9	O	−0.004
10	O	+0.004
11	O	−0.000
12	O	−0.000
13	O	−0.000
14	O	−0.000
Sum		0.000

S8 Canonical Band Structure

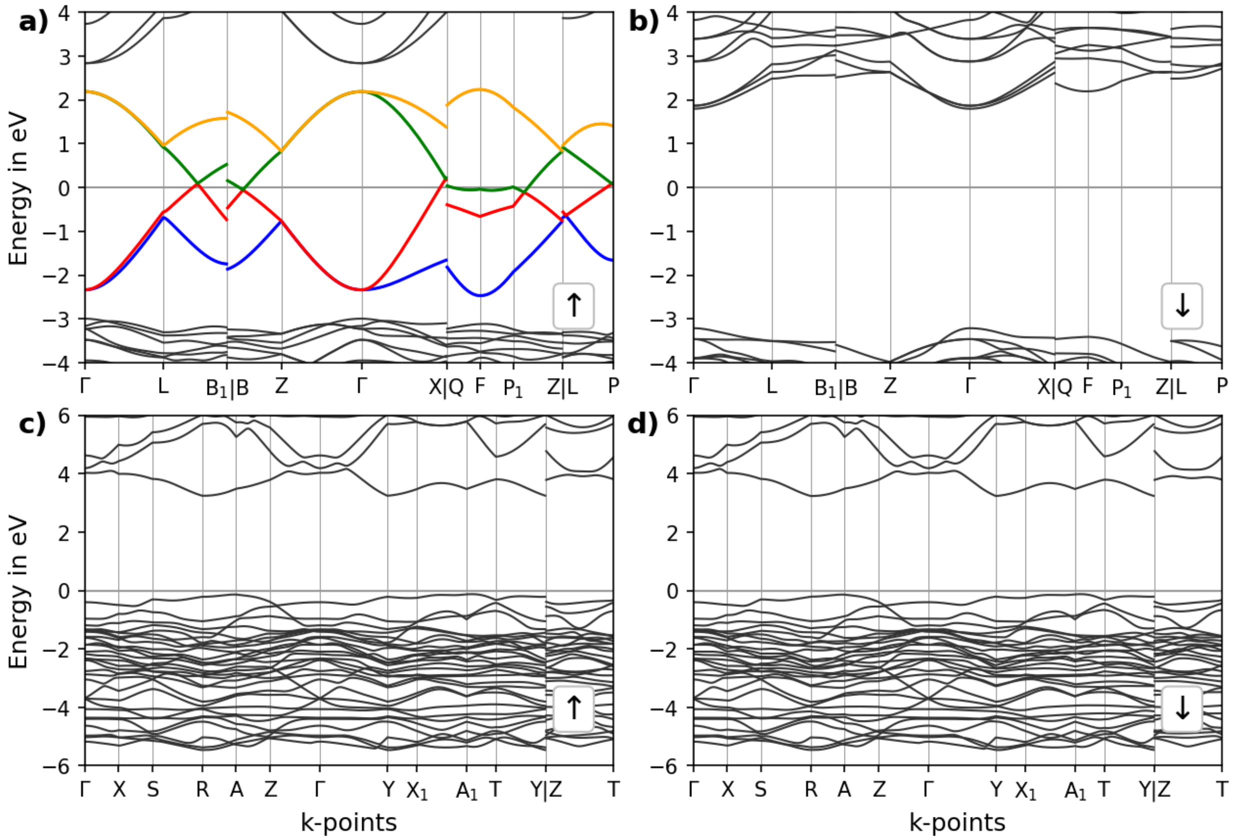

Figure S5: Electronic band structure in canonical path representation for LM (a,b) and LC (c,d) systems. Spin-up and spin-down band structures are shown in a,c) and b,d), respectively. The band path was calculated using the Setyawan-Curtarolo[S10] convention. The Fermi level is set to 0 eV.

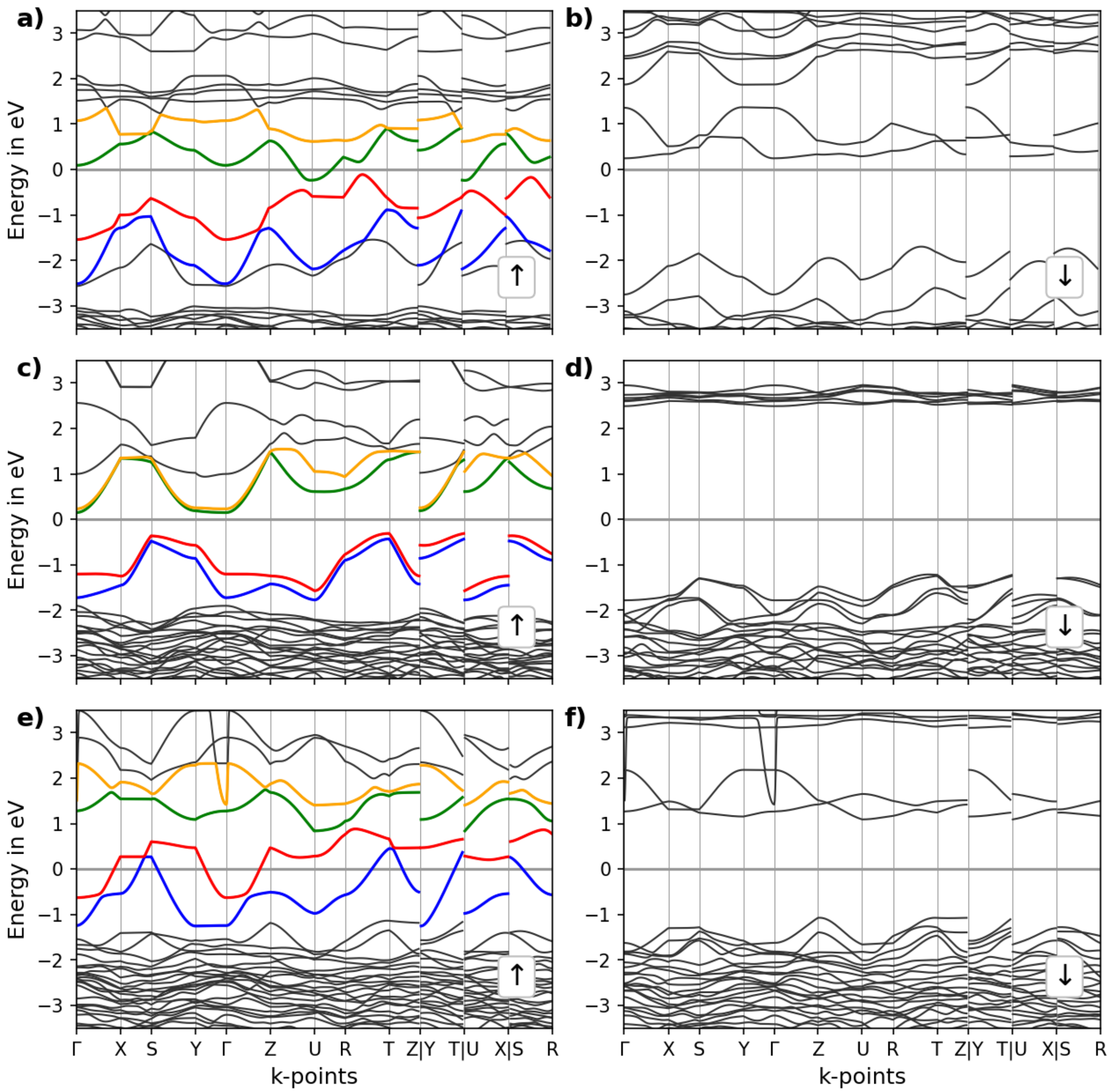

Figure S6: Electronic band structure in canonical path representation for LCM37 (a,b), LCM55 (c,d) and LCM73 (e,f) systems. Spin-up and spin-down band structures are shown in a,c,e) and b,d,f), respectively. The band path was calculated using the Setyawan-Curtarolo[S10] convention. The Fermi level is set to 0 eV.

References

(S1) Mohammadi, A.; Thurner, C. W.; Haug, L.; Bekheet, M. F.; Müller, J. T.; Gurlo, A.; Hejny, C.; Nezhad, P. D. K.; Winkler, D.; Riedel, W.; Penner, S. How defects in lanthanum iron manganite perovskite structures promote the catalytic reduction of NO by CO. *Mater. Today Chem.* **2024**, *35*, 101910.

(S2) Radaelli, P. G.; Jorgensen, J. D.; Schultz, A. J.; Hunter, B. A.; Wagner, J. L.; Chou, F. C.; Johnston, D. C. Structure of the superconducting $La_2CuO_{4+\delta}$ phases ($\delta \approx$0.08,0.12) prepared by electrochemical oxidation. *Phys. Rev. B Condens. Matter* **1993**, *48*, 499–510.

(S3) Åsbrink, S.; Norrby, L. J. A refinement of the crystal structure of copper(II) oxide with a discussion of some exceptional e.s.d.'s. *Acta Crystallogr. B* **1970**, *26*, 8–15.

(S4) Attfield, J. P.; Férey, G. Structure determinations of $La_2O_2CO_3$-II and the unusual disordered phase $La_2O_{2.52}(CO_3)_{0.74}Li_{0.52}$ using powder diffraction. *J. Solid State Chem.* **1989**, *82*, 132–138.

(S5) Rini, E. G.; Gupta, M. K.; Mittal, R.; Mekki, A.; Al Saeed, M. H.; Sen, S. Structural change from Pbnm to R$\bar{3}$c phase with varying Fe/Mn content in (1-x)$LaFeO_3$.x$LaMnO_3$ solid solution leading to modifications in octahedral tilt and valence states. *Journal of Alloys and Compounds* **2021**, *883*, 160761.

(S6) Petrov, A. N.; Zuev, A. Y.; Tikchonova, I. L.; Voronin, V. I. Crystal and defect structure of the mixed oxides $LaMn_{1-z}Cu_zO_{3\pm y}$ ($0\leq z\leq 0.4$). *Solid State Ionics* **2000**, *129*, 179–188.

(S7) Asbrink, S.; Norrby, L. J. CSD 752807: Experimental crystal structure determination. 1970.

(S8) Tuilier, M. H.; Chevalier, B.; Tressaud, A.; Brisson, C.; Soubeyroux, J. L.; Etourneau, J. EXAFS study at the La LIII X-ray absorption edge of superconducting materials obtained by fluorination of $La_{2-x}Sr_xCuO_4$ oxides (0≤ x≤ 0.15). *Physica C: Superconductivity* **1992**, *200*, 113–121.

(S9) Porta, P.; De Rossi, S.; Faticanti, M.; Minelli, G.; Pettiti, I.; Lisi, L.; Turco, M. Perovskite-Type Oxides: I. Structural, Magnetic, and Morphological Properties of $LaMn_{1-x}Cu_xO_3$ and $LaCo_{1-x}Cu_xO_3$ Solid Solutions with Large Surface Area. *J. Solid State Chem.* **1999**, *146*, 291–304.

(S10) Setyawan, W.; Curtarolo, S. High-throughput electronic band structure calculations: Challenges and tools. *Comput. Mater. Sci.* **2010**, *49*, 299–312.
